# Exploring synergistic effects of aerobic exercise and mindfulness training on cognitive function in older adults: Protocol for a pilot randomized controlled trial

**DOI:** 10.1097/MD.0000000000010626

**Published:** 2018-05-25

**Authors:** Elena Salmoirago-Blotcher, Julie DeCosta, Kristie Harris, Christopher Breault, Shira Dunsiger, Claudia Santos, Peter Snyder

**Affiliations:** a Centers for Behavioral and Preventive Medicine, The Miriam Hospital; b Department of Medicine, The Warren Alpert Medical School of Brown University; c Department of Behavioral and Social Science, School of Public Health, Brown University; d Interdisciplinary Neuroscience Program, University of Rhode Island; e Departments of Neurology and Ophthalmology, Rhode Island Hospital and The Warren Alpert Medical School of Brown University, RI, USA.

**Keywords:** aerobic exercise, cognitive function, dementia, mindfulness

## Abstract

**Introduction:**

Despite increasing evidence that aerobic exercise and cognitive training improve cognitive function among patients with cognitive impairment and dementia, few studies have focused on the effect of a combination of these approaches. This study will explore whether combining aerobic training (AT) with mindfulness training (MT), an intervention promoting the moment-to-moment awareness of physical sensations, affective states, and thoughts, improves cognitive function in individuals at risk of dementia. The primary objective is to determine the feasibility and acceptability of the intervention(s). The secondary objective is to obtain estimates of effect sizes on cognitive function and on possible mediators.

**Methods and analysis:**

Forty participants with at least 2 risk factors for dementia will be randomized (2 × 2 factorial design) to either AT (3 sessions/week for 12 weeks), MT (1 session/week for 8 weeks), both, or usual care. Assessments of cognitive function (attention, executive function, episodic, and working memory); physical activity (accelerometry), aerobic capacity (6-minute walk test), waist-to-hip ratio, blood pressure, social support (Multidimensional Scale of Perceived Social Support), depression (Hospital Anxiety and Depression Scale), and mindfulness (Five Facets of Mindfulness) will be conducted at baseline, end of treatment, and 6-months postbaseline. Rates of retention, attendance, and program satisfaction will be calculated for each of the 4 groups to determine the feasibility and acceptability of each intervention.

**Ethics and dissemination:**

This study has full ethical approval by The Miriam Hospital Institutional Review Board and adheres to the Standard Protocol Items: Recommendations for Interventional Trials reporting recommendations. If results from this exploratory, proof-of-concept study support our hypotheses, we will conduct a large randomized controlled trial (RCT) to determine the efficacy of combined MT and AT in improving cognitive function in individuals at risk of dementia. Results from the study will be disseminated through peer-reviewed journals and conference presentations.

**Registration details:**

http://www.clinicalstrials.gov identifier NCT03289546.

## 1 Introduction

In 2014, 5.2 million Americans had Alzheimer disease.^[1]^ Due to longer life expectancy and demographic changes, prevalence rates of dementia are projected to increase nearly 4-fold in upcoming decades. There is currently no effective pharmacological treatment to reduce the risk of dementia and age-related cognitive impairment or improve the disease course.

Although pharmacological treatments are under study, increasing attention has been dedicated to preventive and behavioral strategies. Interest in behavioral approaches stems from findings suggesting that up to a 3rd of Alzheimer disease cases worldwide may be attributable to modifiable risk factors^[2]^ and that improvements in cognitive and physical inactivity in midlife could prevent up to 3 million dementia cases.^[3]^ Strong observational evidence indicates that physically^[4–9]^ and cognitively stimulating^[10–13]^ activity may prevent cognitive decline or dementia. In contrast, evidence from randomized clinical trials (RCTs) is less clear, and reveals that improvements from cognitive training are limited to training-specific domains, with little evidence that benefits generalize to other cognitive capabilities or translate into meaningful improvements in activities of daily living.^[14–17]^ For exercise, findings from RCTs and meta-analyses indicate that aerobic exercise produced small to moderate improvements in cognitive function among patients with mild cognitive impairment and dementia, while no effect has been observed among healthy individuals with no cognitive impairment at baseline.^[18–21]^

Few studies have focused on the effect of combined cognitive and aerobic training (AT).^[22,23]^ A 2 × 2 RCT of cognitive training and aerobic activity found no difference between intervention and control participants but used very active control conditions.^[23]^ A 2nd small RCT suggested that combined aerobic and cognitive training could produce larger effects than either technique alone.^[24]^ Evidence from fMRI imaging studies also indicates that combined approaches may increase brain plasticity.^[25]^

Mindfulness training (MT) is a behavioral intervention designed to increase mindfulness, the “nonjudgmental, sustained, moment-to-moment awareness of physical sensations, affective states, and thoughts.”^[26]^ MT, which is essentially an intensive attention-training intervention,^[27]^ has shown potential to improve attention and working memory by increasing the ability to override irrelevant stimuli.^[28]^ These effects emerged across populations, with RCTs comparing MT to control conditions resulting in improved working memory capacity among middle school and graduate students, as well as military personnel.^[29–31]^ Additionally, experienced mindfulness meditators perform better on assessments of attention compared to controls without meditation experience.^[32]^ Another RCT showed significant improvements in executive functioning in older adults who completed 8-weeks of MT compared to wait list control.^[33]^

Taken together, this evidence supports the potential for a beneficial effect of approaches combining AT and MT. No study thus far has explored the possible cumulative effect of MT and AT on cognitive function. This proof of concept study, titled the Active Minds Study (NCT number 03289546), is proposed in preparation for a future large efficacy RCT with the primary outcome of determining whether the combination of MT and AT is feasible and acceptable. The study will also explore whether a combined MT and AT intervention improves cognitive function, and whether improvements in aerobic capacity, physical activity, cardiovascular risk factors, and mindfulness skills are associated with changes in cognitive function. This work was supported by a grant from the Norman Prince Neurosciences Institute to Drs Salmoirago-Blotcher and Snyder. The Standard Protocol Items: Recommendations for Interventional Trials guidelines were followed in reporting the protocol for this ongoing study.^[34,35]^ Participant recruitment began April 2016 and the study is expected to be completed by December 2018.

## 2 Methods and analysis

### 2.1 Study design and setting

**Figure 1:**
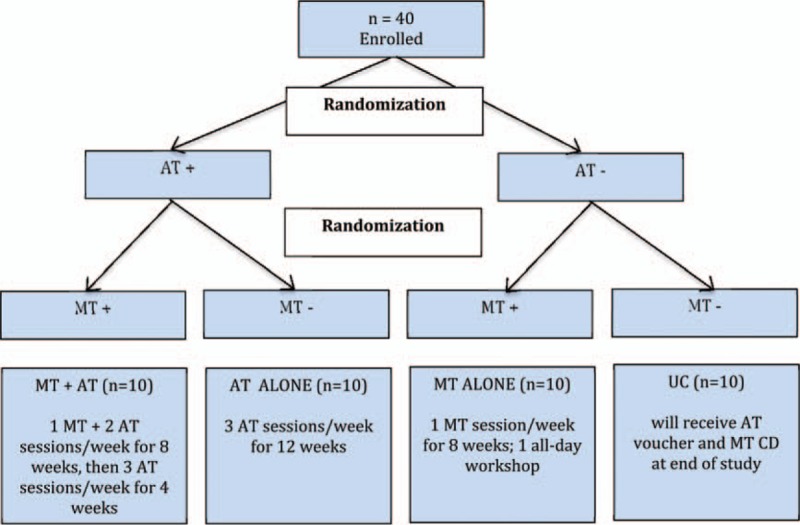
Active minds study design. AT = aerobic training, MT/AT = both conditions (mindfulness training/aerobic training), MT = mindfulness training, UC = usual care.

This study utilizes a 2 × 2 factorial design. Participants are randomly assigned to MT, AT, both treatments, or neither (see Fig. 1). Study assessments and MT sessions are conducted at the Centers for Behavioral and Preventive Medicine at the Miriam Hospital in Providence, RI, while AT sessions are held at a local YMCA.

### 2.2 Population

This study will enroll 40 patients (10 per condition). Inclusion criteria are: At least 2 risk factors for dementia; Age ≥55; Being physically inactive (defined as not meeting current AHA recommendations for physical activity, that is, <150 minutes of moderate-intensity aerobic activity per week OR < 75 minutes of vigorous aerobic activity per week using the 7-day Physical Activity Recall Questionnaire)^[36–40]^; English fluency; and Ability to understand the study procedures and willingness to commit to the demands of the study protocol. Exclusion criteria include: Inability or unwillingness to provide informed consent; Contraindications to physical activity; Blood pressure >200/110 measured at screening visit; Severe depressive symptoms (Hospital Anxiety and Depression Scale depression subscale score >14)^[41]^; History of psychosis; Severe cognitive impairments (Mini-Mental State Examination score < 24)^[42]^; Hospitalization in the previous 6 weeks; and Current (at least once a month) practice of mindfulness meditation, yoga, or tai chi. In addition, the participant's primary care provider (PCP) written permission to participate in the study is required. This study protocol was approved by The Miriam Hospital Institutional Review Board (IRB).

### 2.3 Recruitment

We will have 2 cycles of intense recruitment efforts in order to enroll about 20 participants per recruitment cycle. Recruitment approaches include: Flyers placed in clinics and local public venues (eg, libraries, supermarkets); Advertisements in local newspapers; and Online resources (eg, The Miriam Hospital Intranet, Craigslist, and social media).

### 2.4 Screening and consent procedures

Initial contact is made via telephone by interested individuals using a dedicated phone number included in all recruitment materials. Patients signaling interest are then invited for a screening visit.

Full informed consent and HIPAA authorization is obtained in person by the senior research assistant (SRA) after a thorough explanation of the study design, study intervention, and the risks and benefits involved. After informed consent procedures are completed, participants undergo a battery of screening assessments (see Study Assessments for details).

Following the screening visit, patients enter a 2-week run-in period during which they wear accelerometers for the assessment of baseline level of physical activity and obtain clearance for participation from their PCP. Accelerometers are fitted during the screening visit and returned via mail using a prestamped envelope.

Information is collected from all individuals assessed for eligibility (ie, individuals who consented but then resulted noneligible at the completion of the screening process) in order to provide a complete CONSORT diagram and description of the recruitment process. This information will also serve as an indicator of future generalizability of the study.

Once all screening assessments are completed participants are randomly assigned (1:1:1:1 ratio) to the 4 different conditions. The randomization schedule was generated in “R” (cran.us.r-project.org) and is based on a permuted block randomization scheme with small, random-sized blocks. The allocation table was uploaded to an ACCESS database to conceal the sequence and the SRA performs randomization by clicking the “randomize” button.

Participants are not blinded to treatment allocation, but are blinded to the study hypothesis. For practical reasons, the MT instructor cannot be blinded, however s/he is not be involved in data management or analysis. Blinded personnel include the Principal Investigator, co-investigators, and all data entry and analysis personnel.

### 2.5 Study interventions

Participants assigned to Mindfulness Training (MT) alone receive MT based on the curriculum of the manualized Mindfulness-Based Stress Reduction (MBSR) program created by Jon Kabat-Zinn at the University of Massachusetts.^[26]^ Participants attend an orientation session during which they are introduced to the purpose, content, and schedule of the intervention, followed by one two and a half-hour MT sessions once a week for 8 weeks. The MT protocol includes the following basic components of traditional MBSR programs: training in awareness of sensations (“body scan”), a technique based on the cultivation of attention to bodily sensations that normally go unnoticed; training in the awareness of the sensations of breathing; training in directing the attention to simple activities of daily life, and in recognizing when the attention is no longer focused on a specific object of attention; and training in “open awareness”, a practice in which participants are instructed to just notice to which event (physical sensation, sound, visual object, and thought) their attention is spontaneously drawn from moment to moment. The MT instructor is a graduate of the teachers’ training program at the University of Massachusetts Center for Mindfulness with ≥5 years teaching experience.

In addition to MT sessions, participants are instructed to practice mindfulness techniques for 30 minutes daily on their own with the guidance of a digitally recorded, standardized guided mindfulness practice. The recording is provided in different formats (CD or MP3 file) depending on the participant's preference; CD players are offered to participants who do not possess 1 and MP3 versions are uploaded onto the patient's smartphone or other device.

Participants assigned to aerobic training (AT) alone attend a 1-hour AT session 3 times per week for 12 weeks. Sessions consist of a 10-minute warm-up, 40 minutes of aerobic exercise (walking on a treadmill), and 10 minutes of cool down and stretching. Participants receive heart monitors at the beginning of the study and are trained to exercise targeting heart rates at 65% to 75% of the age predicted max heart rate or at an intensity of 12 to 13 in the Borg scale of the rate of perceived exertion. Exercise sessions are supervised by a Master's level (Kinesiology) instructor.

Participants assigned to the MT + AT condition attend 2 AT sessions and 1 MT session every week for 8 weeks, followed by 3 AT classes per week for 4 additional weeks. Participants are encouraged to participate in as many sessions as possible and are contacted by the SRA each week to remind them of their weekly classes.

Participants assigned to neither intervention continue with the care prescribed by their physicians. To increase retention, at study completion these participants will receive a voucher to attend the YMCA free for 3 months as well as a copy of the MT practice recording.

Although participating in the study, all participants are allowed to continue all medications and treatments (ie, dietary recommendations) prescribed by their care providers. However, participants, regardless of group assignment, are prohibited from engaging in yoga, tai chi, or other mind-body training while enrolled in the study.

To increase overall study retention, patients are asked to provide a home and mobile phone number and email address. The study tracking system identifies participants due for a visit and mail/email reminders or phone messages are then sent to remind patients of their follow-up appointments or class sessions. In addition, we provide a $40 monetary incentive at each of the 3 assessment visits (baseline, 3 month, and 6 month), as well as small gifts such as water bottles and heart monitors. To avoid bias, no incentive is provided to facilitate participation in intervention sessions.

### 2.6 Safety considerations

Both AT and MT are behavioral, low-risk interventions. Aerobic exercise has been shown to be safe in older and deconditioned individuals and in patients with coronary heart disease.^[37–40]^ The most common adverse effects of exercise include muscle soreness (usually mild and lasting only a few days). Less likely effects include dizziness, faintness, fatigue, or falls. Although rare, exercise may also result in serious side effects such as shortness of breath, chest pain, or cardiac arrhythmias. Protection against these risks includes careful screening, adequate supervision during exercise, and instructing patients to recognize untoward signs and symptoms during exercise. The AT instructor is trained in cardiopulmonary resuscitation and an automated external defibrillator is available at the YMCA facility in the rare case of a cardiac event.

Prior to each exercise session participants complete the revised Physical Activity Readiness Questionnaire^[43]^ to determine whether they have experienced chest pain, shortness of breath, dizziness, or joint or muscle injury since the last session.^[44]^ They are also instructed to recognize the occurrence of symptoms such as chest pain, shortness of breath, numbness, and dizziness during exercise. Should a patient report any of these adverse symptoms before or during exercise, any activity will be immediately stopped and he or she will be referred to their PCP and asked to refrain from any physical activity until receiving a complete medical evaluation. If symptoms are severe the patient will be escorted to the nearest Emergency Department for immediate treatment.

Rarely, MT may produce psychological distress.^[45]^ The distress is typically mild, of short duration, and rarely occurs in the absence of serious ongoing psychiatric conditions. To minimize the likelihood of distress, participants with severe depressive symptoms or ongoing psychosis are excluded from study participation. The MT instructor actively inquires about psychological side effects that might occur during the session or individual practice. If a participant presents with signs of severe psychological discomfort, s/he will be excused from the MT intervention and be screened for suicide risk by a clinical psychologist.

Several protections have also been instituted to protect the privacy and confidentiality of participants, including numerical coding, deidentification, and locked storage of all data. Audio recordings of MT sessions are reviewed promptly to ensure treatment fidelity and then destroyed. Instructions for maintaining privacy and confidentiality are provided to participants at the start of their first MT session, as well as reminders at all subsequent sessions.

### 2.7 Study assessments

Assessments are performed at baseline, intervention completion (3 months), and 6 months since baseline (see Table 1). Visits take between 2 and 3 hours to complete and include the completion of study questionnaires, the 6-minute walk test, direct measurements of hip and waist circumference, height, weight, and accelerometer fitting.

**Table 1:**
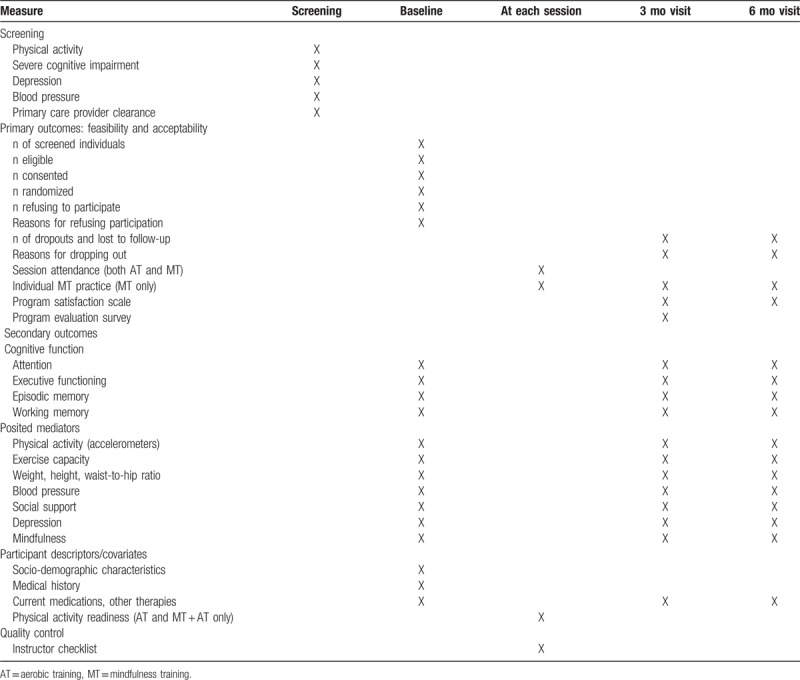
Overview of study measures and timing of assessment.

#### 2.7.1 Screening assessments

The 7-day Physical Activity Recall Questionnaire^[36]^ is used to determine physical activity status for screening purposes. Patients are considered physically inactive if they do not meet current recommendations (<150 minutes of moderate-intensity aerobic activity per week OR < 75 minutes of vigorous aerobic activity per week).^[37–40]^ This measure is also administered at 3 and 6 month assessments as a backup in case of accelerometer failure.

The Mini Mental State Examination^[42]^ is used to screen for severe cognitive impairment, with participants scoring less than 24 excluded from the study.

Blood pressure is measured using a Dinamap XL automated blood pressure monitor according to current recommendations.^[46]^ Participants with blood pressure >200/110 or symptomatic orthostatic blood pressure decrease of >20 mm Hg are excluded from the study and referred to their PCP for follow-up.

Depression is assessed with the 7-item depression subscale of the Hospital Anxiety and Depression Scale.^[41]^ Subscale scores range from 0 to 21, with higher scores indicative of greater depressive symptoms. Participants with depression subscale scores >14 are excluded from the study and referred to their PCP.

#### 2.7.2 Primary outcomes

Measures of feasibility include the number of screened, eligible, consented, randomized participants, retention rates, session attendance, and individual mindfulness practice. Attendance at MT and AT sessions is recorded using an attendance log. Individual mindfulness practice is tracked using a daily self-report log.

A satisfaction survey is used to assess acceptability. The intervention will be considered acceptable if ≥80% of participants respond that they are at least somewhat satisfied with the intervention.

#### 2.7.3 Secondary outcomes

As a secondary goal we will gather preliminary estimates of the effect of group (MT alone, AT alone, both, or neither) on changes from baseline in cognitive function. We expect that participants assigned to the combined MT + AT condition will achieve larger positive changes in cognitive function compared to either condition alone or none.

Participants are administered a battery of cognitive measures to assess attention (Digital Symbol Substitution Test), executive function (F-A-S verbal fluency test), and episodic memory (International Shopping List Test). Scores from these 3 cognitive domains will be used to calculate a single composite score (Z-scores of Attention, Verbal fluency, and Episodic memory for Non-demented adults [ZAVEN]).^[47]^ Working memory is also assessed using 3 measures available from CogState, Ltd, including the Groton Maze Learning Test, One-Card Learning Task, and One-Back Test.^[48,49]^ Each cognitive measure takes approximately 3 to 5 minutes to complete and is administered electronically using a tablet device. All participants undergo a practice trial prior to the administration of cognitive assessments.

#### 2.7.4 Posited mediators

Because there is evidence that RCTs of behavioral interventions based on solid theoretical models are likely to result in larger effect sizes than comparable interventions not based on sound theory, we will explore possible mechanisms by which MT and AT may affect cognitive function. This information will help us to refine the theoretical framework for a future larger RCT.

Accelerometry is used to assess physical activity (Actigraph, LLC, Fort Walton Beach, FL).^[50–52]^ Measures from the accelerometer data include the number of valid days worn, wear time, average step counts per minute, average step counts per day, average minutes of moderate to vigorous physical activity per day, and the average minutes of bouts of this level of activity per day.

The 6-minute walk test is use to assess exercise capacity.^[53]^ This measure is simple, safe, and reliable and performance correlates with peak oxygen uptake.^[54]^

Weight, height, and waist-to-hip ratio are assessed using standardized procedures. A Dinamap XL automated blood pressure monitor is used to measure blood pressure according to current recommendations.

The 12-item Multidimensional Scale of Perceived Social Support^[55,56]^ is used to assess perceptions of support from 3 sources, including family, friends, and a significant other.

Depression is assessed with the 7-item depression subscale of the Hospital Anxiety and Depression Scale (see description in Screening Assessments).^[41]^

Mindfulness is measured using the Five Facets of Mindfulness Questionnaire.^[57]^ This 39-item questionnaire assesses the 5 identified components of mindfulness meditation: observing, describing, acting with awareness, nonjudging of inner experience, and nonreactivity to inner experience.

#### 2.7.5 Covariates and moderators

Information about socio-demographic characteristics including age, sex, race/ethnicity, education, income, and insurance status is collected using standard validated self-report forms. A self-report form is used for participants to record current medications and other therapies. Participant medical history pertaining to coronary risk factors, previous myocardial infarction or stroke, comorbidities, and total cholesterol (mg/dL) history is abstracted from electronic medical records.

### 2.8 Statistical analyses

Our choice of sample size (n = 40, 10 participants per condition) was determined by the scope and timeline of this project. Given that this is a proof of concept study, our focus is not on strict hypothesis testing but rather on estimates of effect size and corresponding confidence intervals.

Depending upon variable characteristics (categorical or continuous), Fisher exact test or *t* tests will be used to compare participants at baseline on socio-demographic and medical characteristics. Baseline characteristics not balanced by randomization, as well as variables associated with the study outcomes, will be included as covariates in linear or logistic regression analyses.^[58,59]^

Rates of retention and attendance, as well as program satisfaction scores at 3 and 6 months will be calculated separately for each of the 4 groups. The study will be considered feasible if retention rates of at least 80% are achieved, if participants attend at least 70% of the planned sessions, and if they report completion of at least 70% of the assigned individual home practice exercises. For acceptability, each intervention will be considered acceptable if at least 80% of participants indicate grade 4 enjoyment ratings on a visual scale ranging from 0 to 4.

To determine effect sizes on cognitive function, the ZAVEN^[47]^ composite cognitive score will be calculated for each participant by converting Digital Symbol Substitution Test, F-A-S verbal fluency test, and International Shopping List Test scores to standardized z-scores. The effects of group on ZAVEN scores at 3 and 6 month follow-ups will be estimated using a series of mixed effects longitudinal models controlling for baseline. Models will include a subject-specific intercept to adjust for repeated measures of the outcome over time within participant. A single model will allow for all pairwise comparisons between groups (MT + AT vs MT alone, vs AT alone, vs neither) with the goal of estimating effect sizes and corresponding confidence intervals. A similar approach will be used to assess effects of intervention on each measure of working memory (Groton Maze Learning Test; One-Card Learning Task; and One-Back Test). Last, exploratory mediation analyses will be conducted to assess associations between changes in fitness and other mediators at 3 months with changes in cognitive score at 6 months. As sample size is limited for mediation models, our interest in is estimating effect sizes for both the *a* path (group effects on mediators at 3 months) and *b* path (mediators at 3 months on ZAVEN scores at 6 months), using a similar set of mixed effects models as described previously.

To account for missing data, analyses will be performed according to the intention-to-treat approach.^[60]^ If participants withdraw from the intervention they are asked to complete follow-up assessments. If substantial missing data are encountered, possible options include using accepted statistical methods (eg, use of complete data only, use of multiple imputation, and use of modified weights and model-based procedures). Sensitivity analyses will be conducted using different assumptions regarding the mechanism of missingness.

### 2.9 Quality control and monitoring

Quality control measures are implemented across 4 study domains, including treatment fidelity, staff training, data monitoring, and metrics. Assessments of treatment fidelity are performed following the guidelines developed by the Treatment Fidelity Workgroup. Auditor checklists were created during the study start-up period and completed during each MT and AT session to monitor fidelity to the intervention. Optimal treatment fidelity would be evidenced by 100% of objectives met. Staff involved in data collection received training in the administration and scoring of all cognitive measures and questionnaires, as well as how to review assessment instruments immediately for omissions.

For data monitoring, quarterly error-checking procedures have been implemented to ensure that all entered data accurately represent data collected. Additionally, once every 12 months an Independent Study Monitor will review study progress and compile a report detailing adverse events and their likelihood of being related to the study intervention, whether adverse event rates are consistent with prestudy assumptions, rates, and reasons for study withdrawal, whether all participants met entry criteria, and whether continuation of the study is justified. This report is then distributed to the Principal Investigator and the IRB.

### 2.10 Ethics and dissemination

Full informed consent and HIPAA authorization is obtained from each participant enrolled into the study. All aspects of the study have received full ethical approval by the IRB at The Miriam Hospital. The IRB will conduct a planned audit of the study every 12 months. Any modifications to the protocol will be submitted as amendments to be reapproved by the IRB. Once approved, the trial registry description (NCT 03289546) will be modified to reflect the changes. Additionally, participants will be reconsented if such modifications include substantive changes to the study procedures or risk/benefit profile. Adverse events experienced by participants are also tracked and reported to the IRB. Given that this is a safe, low-risk pilot behavioral trial, there are no planned interim analyses nor preestablished stopping guidelines.

Results from the Active Minds Study will be disseminated through conference presentations and publication submissions to peer-reviewed journals. An overview of study results will also be posted on the trial registry site at http://www.clinicaltrials.gov. Data generated under the project will be shared as per the NIH Grant Policy and The Miriam Hospital IRB guidelines. After the main findings from the study are accepted for publication, interested researchers may request access to the deidentified data and statistical code for educational and research purposes. All requests will be reviewed by the study Principal Investigator and a data sharing agreement must be finalized between all parties prior to releasing the data.

## 3 Discussion

Increasing attention is being devoted to preventive strategies to reduce the occurrence of age-related cognitive impairment and dementia. Early observational findings suggested that interventions that improve cognitive skills may be beneficial. Such evidence has spurred a boom in mass-market cognitive “brain training” apps. Although these approaches are becoming increasingly popular, improvements are apparently limited to task-specific domains and do not transfer to other cognitive skills. What has been shown to be effective, instead, is aerobic exercise, with several RCTs documenting the effect of AT on improved cognition in individuals with mild cognitive impairment or dementia.^[18–21]^ Despite the well-established benefit of AT and the promise of cognitive training, only 4 studies examined the combined effects of these 2 approaches in patients with cognitive impairment. Overall, results from these studies suggest that combining AT with cognitive training may be beneficial.^[22–25]^

Parallel to this work, studies have attempted to determine whether mindfulness-based interventions may lead to improvements in cognitive function. MT has shown improvements in cognitive skills (ie, attention, working memory, and executive function) in both healthy younger and older adults.^[28–33]^ To the best of our knowledge though, MT has never been used to improve cognitive function among individuals at risk for dementia. Among mind-body approaches, tai chi, a Chinese martial art combining slow, gentle body movements, and meditative practices, has shown great promise for reducing risk of dementia among older adults,^[61]^ but because tai chi incorporates both physical activity and meditative practices, it is difficult to identify the “active ingredient(s)” responsible for such improvements and it remains unclear whether improvements in cognition are the result of changes in attention and concentration skills from MT, vascular changes from increased physical activity, or a combination of the 2.

In light of this empirical evidence, this pilot study seeks to explore the possible synergistic effects of aerobic exercise and MT on cognitive function. This study is novel in that these interventions will be applied to a clinically relevant population, that is, individuals at risk for developing dementia.

### 3.1 Strengths and limitations

This is the first study to investigate the effects of AT, MT, and their combination in older adults at risk for dementia. By utilizing a 2 × 2 factorial design, we will be able to describe the role of AT and MT on cognitive function and to determine if the combination may produce effects greater than either intervention alone.

Many prior studies examining the effects of AT and mindfulness-based approaches on cognition, used singular outcome measures such as the Mini-Mental State Examination to describe overall, global cognitive functioning. Although less demanding on researchers and participants, such an approach precludes a more nuanced understanding of the effect on different aspects of cognition. For this study we chose to administer a battery of measures to assess cognitive function across several domains. This comprehensive approach will help explore whether the posited benefits of AT, MT, and MT + AT are domain-specific or extend across domains. Last, this study is also strengthened by rigorous assessments of treatment fidelity throughout delivery of the interventions.

We acknowledge that the interventions proposed in this study are both time and labor-intensive. Participants are asked to contribute between 3 and 4 hours per week over the course of 8 to 12 weeks, not including travel time or time taken to complete study assessments. This schedule was chosen to ensure that participants receive a strong enough “dose” of each intervention and to be as consistent as possible with the structure of the MBSR program.

Detailed feedback will be obtained from participants regarding the length, structure, and quality of all aspects of the MT and AT interventions, and their input will be carefully considered in determining the format of the interventions that will be used in future studies. One possible modification could be to deliver MT using a mobile app instead of requiring participation in class-based sessions, which has the potential to significantly reduce participant burden. Another limitation is the small sample size of this study, as such, results will be only preliminary, and will be used to inform a larger RCT designed to establish the efficacy of this approach.

## 4 Conclusion

If the findings from this pilot trial support the feasibility and acceptability of the MT and AT interventions, we anticipate conducting a fully powered RCT to examine whether MT only, AT only, and MT + AT result in meaningful improvements in cognitive function among patients at high-risk for dementia. Preliminary findings from our exploratory mediation analyses will be used to develop a theoretical framework explaining the mechanisms through which AT and MT, might exert their effect on cognitive function. This framework, along with feedback provided by participants, will be used to design the structure, timing, and delivery of the interventions and assessments used in the larger RCT. Findings that combined AT and MT training is effective in improving cognitive function in individuals at risk of dementia could have enormous impact on the prevention of dementia and age-related cognitive decline.

## Author contributions

**Conceptualization:** Elena Salmoirago-Blotcher.

**Funding acquisition:** Elena Salmoirago-Blotcher, Peter Snyder.

**Investigation:** Elena Salmoirago-Blotcher.

**Methodology:** Elena Salmoirago-Blotcher, Shira Dunsiger.

**Project administration:** Elena Salmoirago-Blotcher, Julie DeCosta.

**Software:** Christopher Breault.

**Supervision:** Elena Salmoirago-Blotcher.

**Writing – original draft:** Elena Salmoirago-Blotcher, Julie DeCosta, Kristie Harris.

**Writing – review & editing:** Elena Salmoirago-Blotcher, Kristie Harris, Peter Snyder, Shira Dunsiger.
